# Supplementary figures and images for: Cytoskeleton polarity is essential in determining orientational order in basal bodies of multi-ciliated cells
Source: PLoS Comput Biol. 2020 Feb 21;16(2):e1007649. doi: 10.1371/journal.pcbi.1007649 (PMC7055923; doi:10.1371/journal.pcbi.1007649)

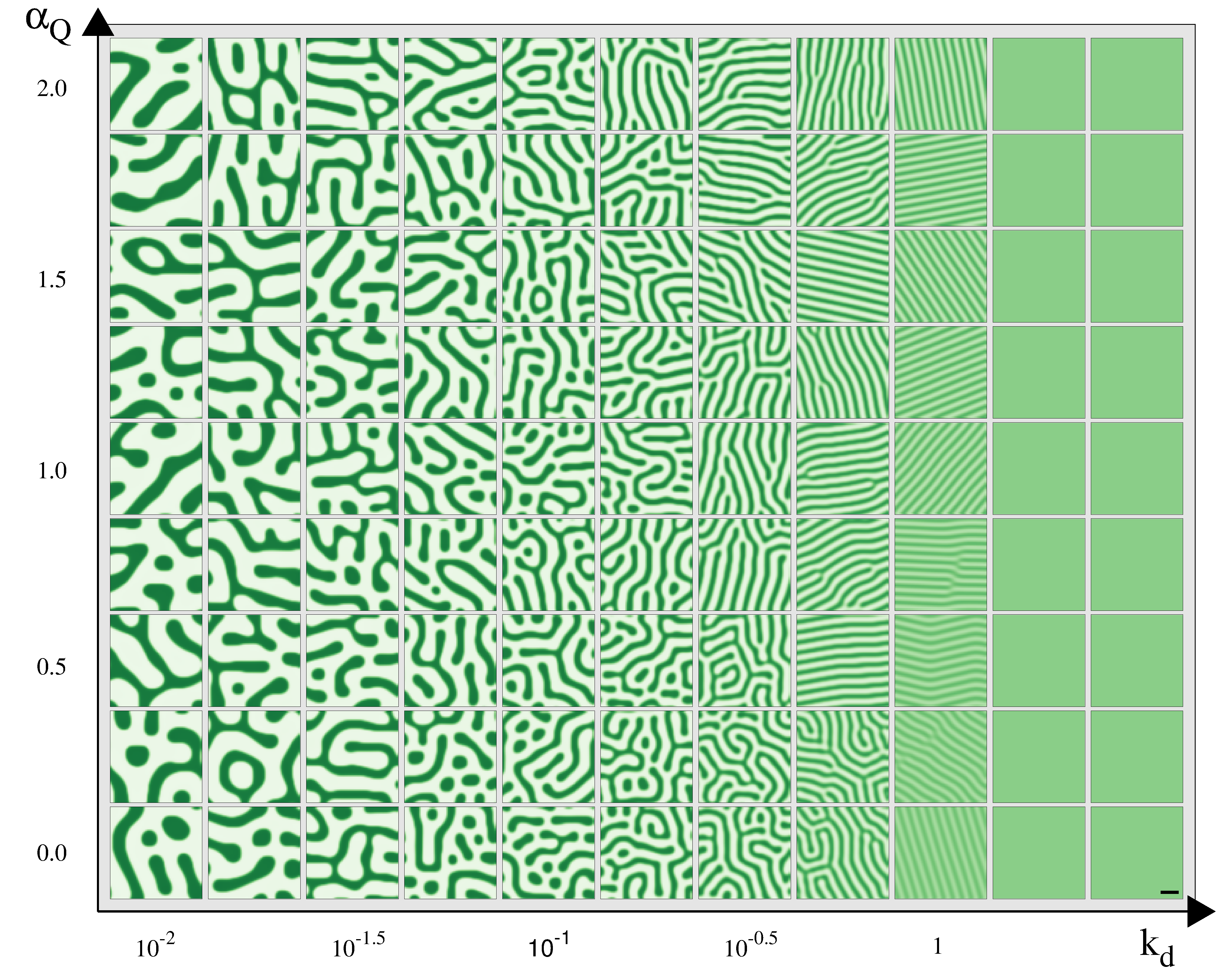

Supplement: S1 Fig — The patterns are dependent on αQ and kd; Striped pattern appears for finite value of αQ (striped patterns which are not sharp may appear at αQ = 0 in a narrow parameter region). CSKs concentration becomes uniform at very high values of polymerization/depolymerization rate kd. Bars, 0.5 μm. (TIF) [file pcbi.1007649.s001.tif]

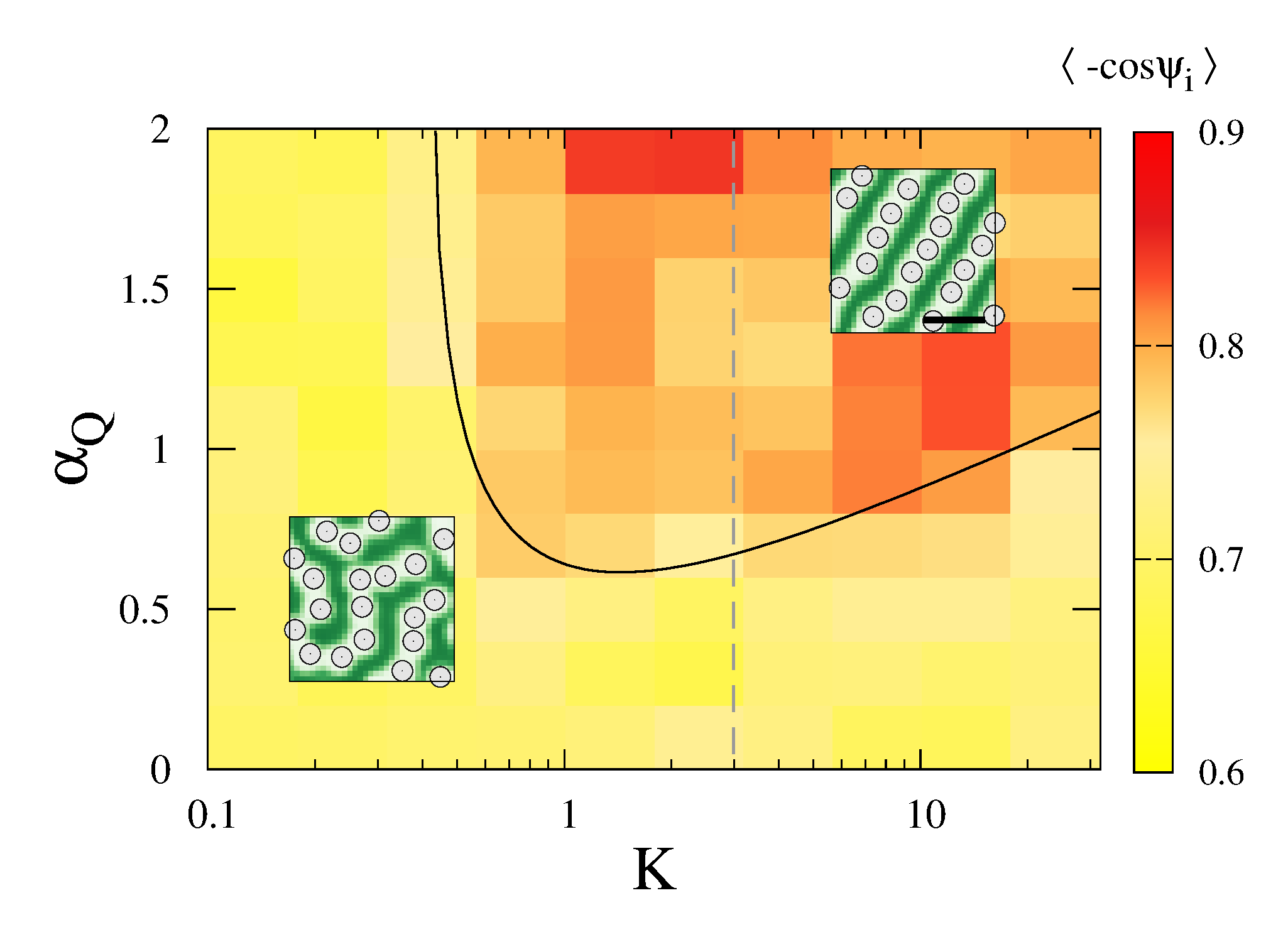

Supplement: S2 Fig — Color indicates the straightness of BB quantified by 〈−cosψi〉. Inset figures show typical pattern of BBs. Gray dashed line (K = 3.0) corresponds to the line connecting between symbol A and PA in Fig 3. Bars, 0.5 μm. (TIF) [file pcbi.1007649.s002.tif]

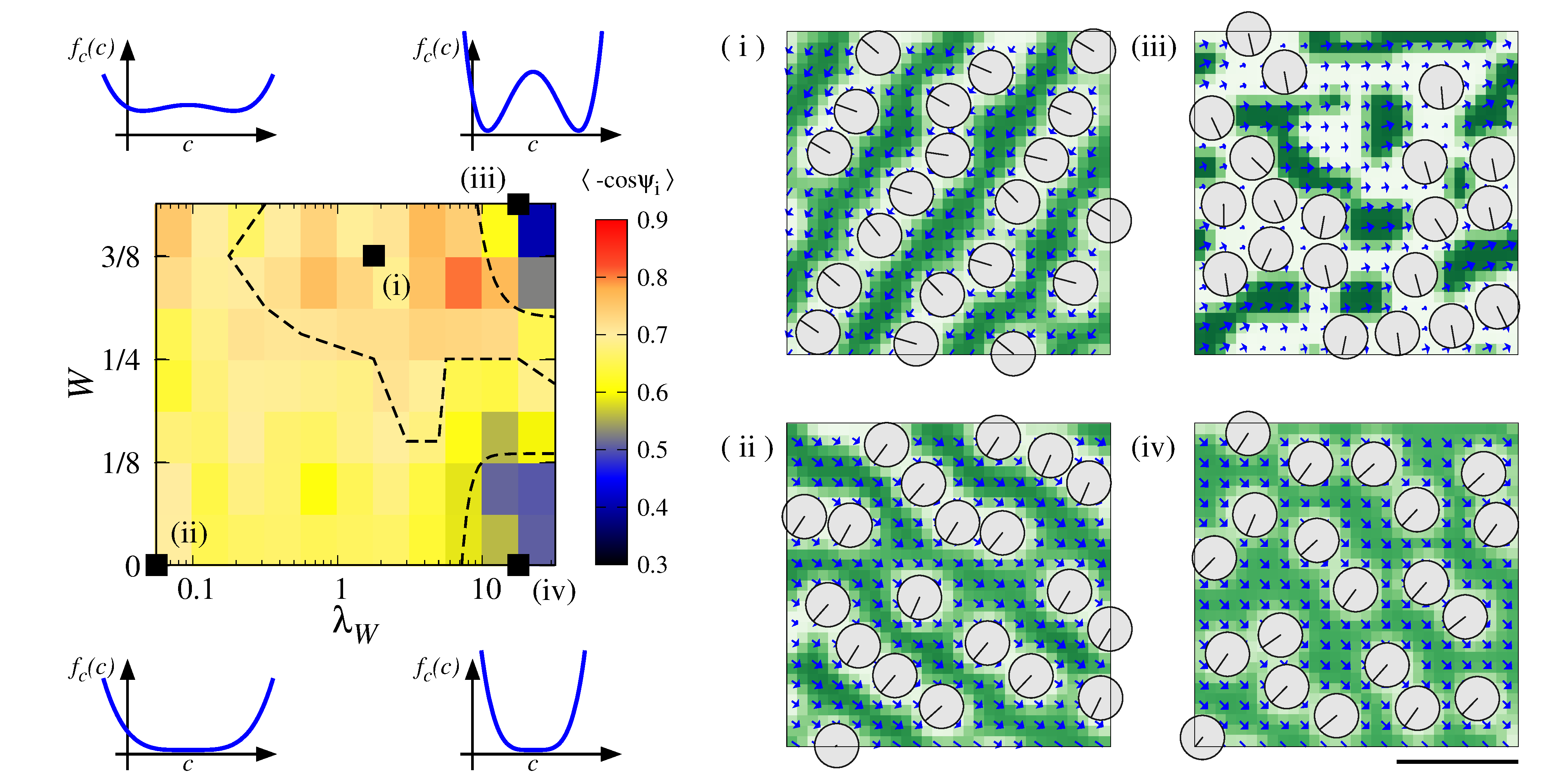

Supplement: S3 Fig — The form of fc is expressed as fc(c) = λW(c−c0−W)2(c−c0+W)2. Left: phase diagram against λW and W. Color indicates the straightness of BB (〈−cosψi〉). Right: Four representative patterns are shown (i-iv) with corresponding black squares in the left panel. Bars, 0.5 μm. (TIF) [file pcbi.1007649.s003.tif]
