# Supplementary material for: Cytoskeleton polarity is essential in determining orientational order in basal bodies of multi-ciliated cells
Source: PLoS Comput Biol. 2020 Feb 21;16(2):e1007649. doi: 10.1371/journal.pcbi.1007649 (PMC7055923; doi:10.1371/journal.pcbi.1007649)
Supplement: Attachment — Submitted filename: PLOScomments.pdf [file pcbi.1007649.s009.pdf]

Summary:

For the cilia of epithelial cells to beat synchronously, the basal bodies have to be arranged along arrays perpendicular to the cell polarity. The cytoskeleton and cell polarity are both responsible for this organization. The paper is about two kinds of order arising in Basal Bodies (BB's) on the apical membrane of epithelial cells- positional and orientational - these are both closely linked to the corresponding Cytoskeletal (CSK) organization at the membrane. The authors present an easy to understand model- a set of dynamical equations for concentration and orientational ordering of CSK's, and position and angle of the Basal Body.

Main Findings:

- In an earlier paper by one of the main authors, the polarity of CSKs was not considered. BB arrays could nevertheless form because the CSKs could condense to form a striped pattern. In this paper the authors also consider the orientation of the BBs. They argue that polarity of the MT bundles must be considered in order for the BB arrays to be orientationally ordered.
- All features of the model have been explored, and the authors provide explanations for clusters, branched arrays, regular straight arrays of BBs and separated BBs.
- They show how excluded volume effects between MTs and BBs are sufficient to produce aligned arrays of BBs. In an earlier model, excluded volume was not considered and the MTs had to spontaneously phase condense into a striped pattern to in order to get the required BB arrangement.
- They also have an explanation for how the BB arrays are aligned perpendicular to the tracheal tract by using boundary conditions appropriately.

Overall comments about the quality:

It is a scientifically interesting paper. The model is methodically explored. It is a good improvement over the authors' previous work on the same subject. The explanations are thoughtful and there is sufficient attention to detail.

The model used is self consistent and makes intuitive sense. Some of the results are not so intuitive and so bring something new to the table: It is also a good quality, thoroughly conducted and well presented computational model, and should be published for its epistemic value.

The work is also novel and this should be better emphasized in the main text: BB's are treated like particles, while MTs have associated concentration and polarity fields. Incorporating a position variable into a free energy functional (usually of 2D fields) is not very often seen. Although the authors give a reference to a similar method used in liquid crystals [17], it may be useful to emphasize this in the abstract/key words.

The main area for improvement is in communication. The authors can considerably improve the communication of certain key ideas. In particular the terms used for different effects are non standard and it will be better to stick to standard liquid crystal jargon. (refer detailed comments).

It meets the standards of PLOS Computational Biology and I recommend it for publishing, contingent on rebutting some of the issues laid out in the detailed comments.

Detailed comments:

1. The title of the paper refers to “direction” of Basal bodies. Not clear whether this refers to the aligned arrays of BBs or orientation of BBs. A more impactful title could be -”CSK polarity is essential in determining orientational order in BBs…”
2. Abstract can include a sentence on what method is used- like “We use a --- method that models BBs as particles in a field of CSKs..”
3. The use of the terms “Alignment” and “Orientation” throughout the text is confusing. Alignment and Orientation are used as the terms to describe the two separate effects: BB arrays forming straight arrays and individual BB’s having a particular orientation. However, these two words mean the same thing so it can be misleading, and needs the reader to keep constant track of which effect is being talked about. Positional and orientational order may be better terms to use.
4. Line 112: Add a reference to the experimental paper.
5. Line 130: Use either orientation of direction, and let the jargon be the same throughout the text.
6. Line 132: “concurrently” can be replaced by - “at the same time”.
7. Why does F_{c\phi} have \phi c^2 and not just \phi c, even though the latter is a lower order effect, not disallowed by symmetry?
8. Line 216: the redefined kd (kp, rescaled by depolymerization rate) should be cross referenced with the entry in the table. Later on this causes confusion in line 258 and 261.
9. Lines 260-263. The fact CSKs can surround BB’s to separate them is interesting. But it is not the excessive amounts of CSK’s, as mentioned in the text, but rather the high turnover that seems to make this possible. The explanation given in the text also conflicts with the fact that the parameters are chosen such that the total concentration of CSKs is constant.
10. Fig 3 and associated text: Given that the turnover time is an important parameter, could the phase C become the phase S, given long enough time? In other words, is it possible that C is a transient phase and at long times, one always gets S? Without getting into the details of dynamics towards steady state, it would help if the authors are reasonably convinced by their simulations that they have genuine steady states. If such checks have been performed, it would be good to mention it in the supporting information.
11. Line 279: Maybe better to just call K “bending modulus”. In the table it is called “propagation of polarity” and in the text line 279, it is called “strength of the polarity”. Once again, a case of non uniform and non standard jargon.

12. Line 283: Different notation is desirable for cos<\phi_i> where in this context \phi_i is a local angle measure the straightness of the BB arrays and in equations 5-7, a variable that gives the concentration of BBs.
13. Line 302 onwards. It is interesting to know that excluded volume effects are enough to attain BB alignment and even give rise to orientational order to some extent, and that there is no need for strong bundling Lower bundling merely shifts the point to another region in the phase diagram. (Just a comment, no corrections)
14. Give a reference for line 333 (If there is one), where BBs align in the absence of bundling proteins.
15. Line 335 onwards: The model for Nocadazole treatment ignores both polarity and phase condensation of CSKs. This gives rise to clusters of BBs, where the clustering is driven by minimizing the interface between BBs and CSKs. Does the cluster size grow over time to further minimize the interface in the tissue and/or simulation?
16. A question about the simulation: For most of the phase diagrams, periodic boundary conditions are used. So the finiteness of the cellular boundaries don’t play much of a role in the patterning of CSKs. So are we to imagine that the simulation actually works across a tissue and cellular boundaries are unimportant? If so, this could improve the explanation of BB alignment across the tracheal tissue talked about in lines 352 onwards.
17. The BB alignment through PCP can be better explained. Why is changing the boundary conditions significant? In Fig 5, why are the boundary conditions made to change suddenly? It seems from the snapshots 3 and 4 in Fig 5 that the transmission of polarity of CSKs takes place very quickly over the length of the tissue. If one is to get a better idea of what is happening, perhaps the system size should be increased along the length of the tissue. If the authors plan to address this in some following work, it should be mentioned.
18. Newman boundary conditions should be changed to Neumann boundary conditions.
