# Supplementary material for: Cytoskeleton polarity is essential in determining orientational order in basal bodies of multi-ciliated cells
Source: PLoS Comput Biol. 2020 Feb 21;16(2):e1007649. doi: 10.1371/journal.pcbi.1007649 (PMC7055923; doi:10.1371/journal.pcbi.1007649)
Supplement: Attachment — Submitted filename: Response_to_reviewers.pdf [file pcbi.1007649.s010.pdf]

Answers to Reviewer 1

The manuscript by Namba and Ishihara deals with the fascinating question of how cilia in a multiciliary array become coordinately polarized. The authors provide a mathematical model for how this is achieved that relies heavily on the interactions between basal bodies and the microtubule cytoskeleton. The authors do an admirable job of summing up the relevant literature and there is certainly some merit to their model. However, I have several serious concerns that warrant consideration.

Thank you for your critical and useful comments on our study. We agree that our model is not perfect and some important aspects could be addressed further. We did not include any analysis on the nucleation of MTs from BF. However, here we have addressed this issue as point-to-point answers to your comments. In the current literature, there are many contradicting experimental interpretations while there are few mathematical models. We would like to restrict our present study to exploring the role of apical MTs and its polarity, which would require a simple and minimum model . We believe that our findings elucidate the possible mechanism of ciliary pattern formation, which might be useful for designing future experiments (we are already collaborating with an experimental group). We have added relevant text in the Discussion of the revised manuscript, explaining the assumptions of our model and have also speculated on problems that could be solved in future based on current experimental observations. This would help the readers to better understand the current literature, and would further stimulate the development of more sophisticated (and alterative) models.

Main questions

1. MT bundles can be parallel or anti parallel. The information in the literature would suggests that these MT bundles are mixed or anti parallel, but the current equations if I understood them correctly rely on parallel bundles all going in the same direction. This is not likely to be the case. At the very best there will be a slight bias in the directionality of the MTs. I think this mixed array needs to feature more heavily into any equations.

Theoretically, macroscopic description of mixture of parallel and anti-parallel MTs in bundles can be expressed using polar and nematic variables, where the nematics indicates equally mixed bundles and polarity indicates the bias in the directionality. Technically, both polarity and nematics can be described by polar variables (like tensor Q in our model). In the case of our model,

polarity is required for directing BBs (process (v)), while the other processes ((i) – (iv)) are based on nematic nature of MTs. Variable *p* in our model can represent both nematic and polar nature depending on context (in other words, *p* does not necessarily indicate fully polarized bundle). This kind of formulation is common in liquid crystal theory [P.G. De Gennes and J. Prost, 'The Physics of Liquid Crystals']. We think that our choice of this model variable is appropriate. Inclusion of additional variables could be useful for more finely distinguishing between parallel and anti-parallel mixture of MT bundles. But for the present study, we would like to keep just a polar variable for the minimal description of the model. We have added an explanation in the revised manuscript for avoiding misunderstanding.

2. Similar to above the authors state "Although more direct evidence of the presence of polarity in MT bundles is still lacking, this can be investigated and confirmed in future experiments." Multiple groups have tried to do this in multiple systems. There is simply not a strong directional bias to the MT orientation before BB polarization. Vladar et al. 2013, Boutin et al 2014, Kim et al 2018 all show some polarized accumulation of MTs in MCCs but this comes after BB polarization and likely reflects the bias in orientation of MTs nucleating from the BF that are all pointing in the same direction. If oriented MTs were defining the polarity for BB then it would come first, which does not seem to be the case. Additionally, all of the mentioned data have polarized subset of MTs parallel with the axis of orientation (and primarily at the cortext) whereas this model is based on the pool of MTs interacting with BBs which must be more perpendicular to the axis of orientation

First, our model is involved with untyrosinated MTs running in parallel with and between arrays of BBs, based on EM images [Kunimoto et al. (Cell , 2012), Herawati et al. (J Cell Biol, 2016), Tateishi et al. (Sci Rep, 2017)]. The main interest of this study is self-organization driven by MTs. To the best of our knowledge, no study has directly investigated direct whether these MTs sustain polarity or not. Second, as you pointed out tyrosinated MTs show asymmetric accumulation along the cellular cortex in later maturation stage [Vladar et al. (Curr Biol, 2012), Boutin et al. (Proc Natl Acad Sci USA , 2014), Kim et al. (J Cell Biol, 2018)]. These two kinds of MTs (untyrosinated bundled MTs and cortical MTs) could be related with each other, such that establishment of a BBs pattern and accumulation of the cortical MTs would proceed simultaneously. This process was not included in our model. The cortical MTs were introduced just as a boundary condition. We suggested a possible mechanism of how the self-organized pattern of BBs by the untyrosinated MTs is modulated by PCP. This is only the first step towards incorporating the full interaction between the apical MTs and PCPs in the mathematical model. We agree that this part

in the model is not sufficient and should be further studied. But we think it is independent of our main findings. For better clarity, we have added further explanation for this point.

3. The authors state "However, this binding between BF and MTs allows BBs to associate with either of the two neighboring MT bundles and hence they cannot distinguish between the two opposite directions (Fig 1C (i)). Therefore, how BBs can determine their definite direction remains a mystery." If I understand things correctly, the authors are treating the MTs and the BB independently. Figure 1C suggests that the BF are interacting with the MTs, which is somewhat consistent with the EM literature. However, what I think is missing from this discussion is where the MTs are originating. I think there is strong indications in the literature that the MTs are in fact at least in part nucleating from the BF. Therefore it is not really appropriate to consider these things completely independent of one another.

Thank you for this comment. As we mentioned in the manuscript, we have also recognized that BF contains MTOC and that MT could be nucleated from BF. When we started constructing the present model, we have considered this possibility. However, we have not found any decisive experimental evidence suggesting that MTs are only nucleated from the BFs, particularly in mouse tracheal cells. Hence, we did not include the process in the model. However, since polymerization of MTs takes places through the apical membrane wherever MT plus-ends exist in the cell (that is our model assumption), we have speculated that the qualitative nature of the model behavior would not change by the details of the MT nucleation mechanism. To further validate this, we performed additional numerical simulations in which MT was created only at the position of BF, as an extreme case (see attached figures for details). We found that BBs are aligned again, against the change of the nucleation manner, confirming that the pattern of BBs was hardly influenced. In addition, upon considering your suggestions, we have started working on the more detailed mechanism of the BBs ordering process (v). In our model, we assumed that direction of BBs follows the orientation of MTs and the detailed process was not considered. Nucleation of MTs at BF might be key for addressing this problem and we could make a more detailed model. However, we think it is beyond the scope of the present study and the dynamics of our present interest (cell scale pattern of BBs) would not change. We have discussed these points in the revised manuscript.

4. Related to above all MTs in this manuscript are created equal which is almost certainly a critical oversimplification. The evidence from Vladar et al. would suggest that there is a

distinct pool of MTs labeled with tyrosinated tubulin that interact with cell cortex in a polarized manner. However the majority of MTs are not tyrosynated in these cells.

As mentioned above, our model mainly describes untyrosinated MTs, and polymerization itself can take place through the apical membrane.

5. Finally and perhaps most concerning is the lack of integration of the key information that hydrodynamic forces are critical to the polarization of these BBs. While the authors mention this in their discussion it is completely lacking from the model. We know from multiple systems that if cilia motility is disrupted (with the rest of the MT cytoskeleton intact) then BBs will not orient. This tells us that the MT BB interactions alone are not sufficient to drive orientation. While I appreciate that this is a complicated question and the connection of MTs and BBs is an important aspect, a model that does not explain the basic experimental evidence needs improvement.

We agree that the hydrodynamic forces are important for enhancing the orientational order formation. However, as we mentioned above, our model was not intended to be perfect that is able to explain all the experimental observations. As we wrote in the Discussion section, only hydrodynamic forces cannot determine the direction, but it could work as a 'back up' for enhancing orientation and synchronization of the ciliary beating. In this study, we separated the problems and focused on only the role of apical MTs and PCP system. We believe that this is a reasonable approach. We hope that we can integrate the problems in future.

Minor:

"In particular, the attachment of BF to MTs might be important for the determination of BB direction, but its mechanistic details have not been identified as yet." I more or less agree with this statement and also feel that in general the authors have done a thorough job of referencing the literature, however I think referencing the Turk et al. paper on gamma tubulin at the basal foot might be appropriate as this is probably the closest we have seen to mechanism for nucleating MTs from the BF.

Thank you for letting us know about the study. We added Turk et al. (Current Biology, 2015) in the revised paper.

Again, we appreciate your comments.

We tried to reflect them in our revised manuscript, and we hope they meets your requirements.

Numerical simulations in which MT was created only at the position of BF

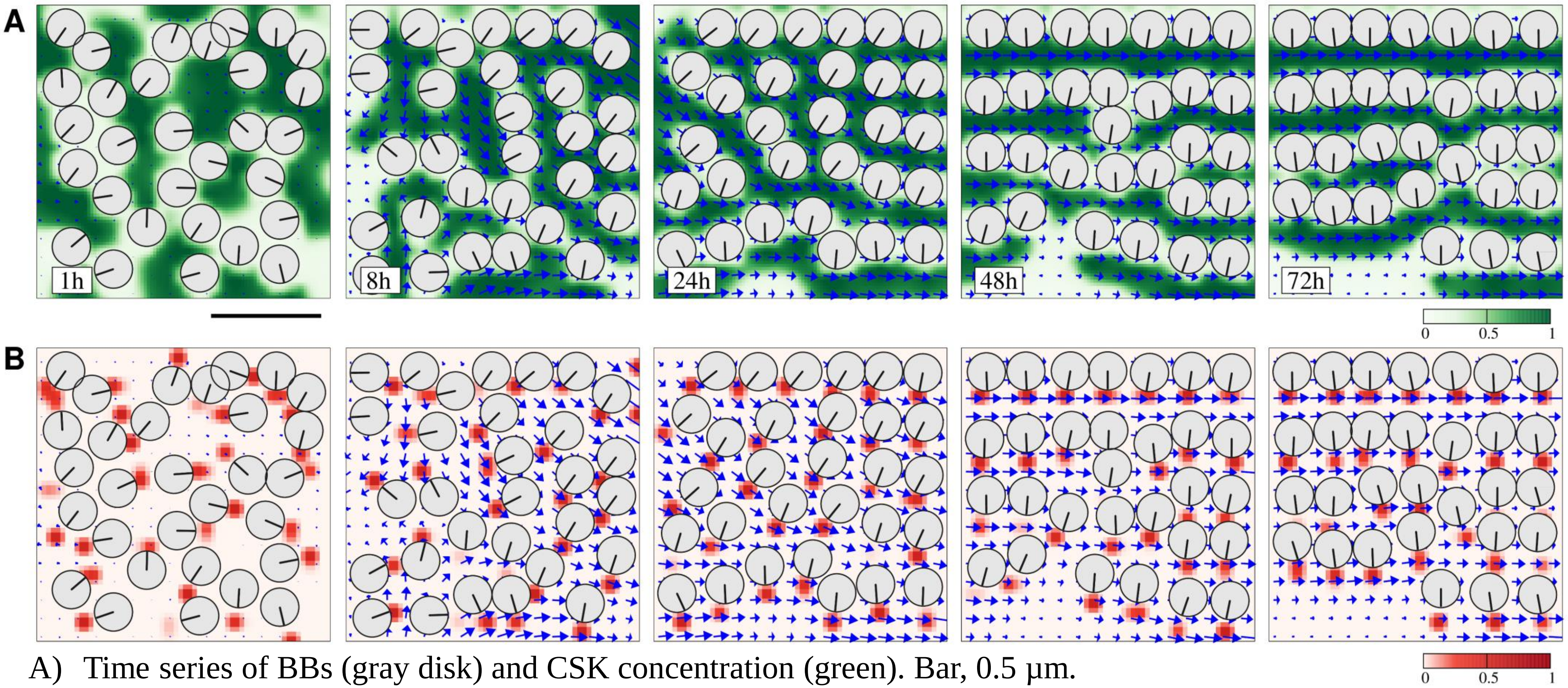

A) Time series of BBs (gray disk) and CSK concentration (green). Bar, 0.5 µm.

B) Region of MT creation ψ_i (red). Time points are same as panel A. Neumann conditions are applied for the boundary.

Points changed from presented model

$$\partial_t c = \nabla \cdot \left(\alpha_J \frac{\delta F}{\delta c}\right) + k_p \underline{\psi_i} - k_d c \quad ,$$

$$\psi_i = \frac{1}{2}\left(1 + \tanh\left(v\left(R_{BF} - \left|\vec{r} - \left(\overrightarrow{X_i} + (R + R_{BF})\begin{pmatrix}\cos\theta_i \\ \sin\theta_i\end{pmatrix}\right)\right|\right)\right)\right)$$

ψ_i : region where MTs are created by BF (red region in panel B)

R : the radius of BB.

R_{BF} : the radius of the region ψ_i.

θ_i : the orientation of BF.

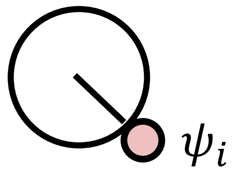

Answers to Reviewer 2

Thank you for your positive and useful comments on our study. We revised the manuscript following your comments, details of which are listed below.

Detailed comments:

1. The title of the paper refers to "direction" of Basal bodies. Not clear whether this refers to the aligned arrays of BBs or orientation of BBs. A more impactful title could be -"CSK polarity is essential in determining orientational order in BBs…"

Title was changed to "Cytoskeleton polarity is essential in determining orientational order in basal bodies of multi-ciliated cells".

2. Abstract can include a sentence on what method is used- like "We use a --- method that models BBs as particles in a field of CSKs.."

We added the following sentence in the abstract. "For considering the excluded volume effect between BBs and CSKs, we used a phase-field method for representing BBs as particles in a field of CSKs."

3. The use of the terms "Alignment" and "Orientation" throughout the text is confusing. Alignment and Orientation are used as the terms to describe the two separate effects: BB arrays forming straight arrays and individual BB's having a particular orientation. However, these two words mean the same thing so it can be misleading, and needs the reader to keep constant track of which effect is being talked about. Positional and orientational order may be better terms to use.

We changed the notation as "positional and orientational orders" following your suggestion.

4. Line 112: Add a reference to the experimental paper.

We add references Herawati et al. (J Cell Biol., 2016) [3] and Kunimoto et al. (Cell, 2012) [9] .

5. Line 130: Use either orientation of direction, and let the jargon be the same throughout the text.

In the revised manuscript, we use 'direction' for the cilia beating and MT polarity, and 'orientation' for BBs. We hope these improve the readability of the manuscript.

6. Line 132: "concurrently" can be replaced by - "at the same time".

We changed it as suggested.

7. Why does $F_{c\phi}$ have ϕc^2 and not just ϕc, even though the latter is a lower order effect, not disallowed by symmetry ?

We can find similar patterns using $F_{c\phi} = \phi c$ by selecting $\lambda_{c\phi}$ and $\lambda_{\phi\phi}$ appropriately. Technically, $F_{c\phi} = \phi c^2$ was chosen for numerical convenience where the BBs are more susceptible to perturbation when the concentration was low. We have added these comments in the "Method" section.

8. Line 216: the redefined k_d (k_{p}, rescaled by depolymerization rate) should be cross referenced with the entry in the table. Later on this causes confusion in line 258 and 261.

We made these changes in the revised manuscript following your suggestion.

9. Lines 260-263. The fact CSKs can surround BB's to separate them is interesting. But it is not the excessive amounts of CSK's, as mentioned in the text, but rather the high turnover that seems to make this possible. The explanation given in the text also conflicts with the fact that the parameters are chosen such that the total concentration of CSKs is constant.

Thank you for pointing this out. As you mentioned, our explanation was not correct. We have corrected the description as "since the amounts of CSKs surrounding BBs recover quickly to the mean concentration".

10. Fig 3 and associated text: Given that the turnover time is an important parameter, could the phase C become the phase S, given long enough time? In other words, is it possible that C is a transient phase and at long times, one always gets S? Without getting into the details of dynamics towards steady state, it would help if the authors are reasonably convinced by their simulations that they have genuine steady states. If such checks have been performed, it would be good to mention it in the supporting information.

Thank you for the advice. We think that the patterns are not transient but are in steady state. The simulation time is much longer than turn-over time, indicating that the turn-over kinetics dose not dominate the time-scale of the pattern formation. To check it, we performed simulations 10 times longer than the original ones and found that the patterns remained unchanged.

11. Line 279: Maybe better to just call K “bending modulus”. In the table it is called “propagation of polarity” and in the text line 279, it is called “strength of the polarity”. Once again, a case of non uniform and non standard jargon.

We adopted “bending modulus” for *K*.

12. Line 283: Different notation is desirable for $\langle -\cos\phi_i \rangle$ where in this context ϕ_i is a local angle measure the straightness of the BB arrays and in equations 5-7, a variable that gives the concentration of BBs.

We changed ϕ_i into ψ_i in the main text, Fig. 4, Fig. 5, Fig. S2, and Fig. S3.

13. Line 302 onwards. It is interesting to know that excluded volume effects are enough to attain BB alignment and even give rise to orientational order to some extent, and that there is no need for strong bundling Lower bundling merely shifts the point to another region in the phase diagram. (Just a comment, no corrections)

Thank you for your comment. Yes, we were also excited and were interested when we found this simulation result. We think that this is one of interesting findings of this study from a theoretical point of view.

14. Give a reference for line 333 (If there is one), where BBs align in the absence of bundling proteins.

We added references Werner et al. (J. Cell. Biol., 2011) [14] and Guirao et al. (Nat. Cell. Biol. 2010) [30].

15. Line 335 onwards: The model for Nocadazole treatment ignores both polarity and phase condensation of CSKs. This gives rise to clusters of BBs, where the clustering is driven by minimizing the interface between BBs and CSKs. Does the cluster size grow over time to further minimize the interface in the tissue and/or simulation?

Thank you for the interesting comment. In the experimental data in MTEC multi-ciliated cells, clusters of BBs formed by nocodazole treatment are typically about 4 μ m in diameter. Once they were formed, we did not observe their fusion, or single cluster of BBs (see Video 8 in Ref. [3]). Moreover, in the simulation, we have not observed the change of cluster size in the range of simulation time, which could be very long. However, the coarsening of clusters is interesting, and we think it is possible.

16. A question about the simulation: For most of the phase diagrams, periodic boundary conditions are used. So the finiteness of the cellular boundaries don't play much of a role in the patterning of CSKs. So are we to imagine that the simulation actually works across a tissue and cellular boundaries are unimportant? If so, this could improve the explanation of BB alignment across the tracheal tissue talked about in lines 352 onwards.

As you pointed out, the periodic boundary condition was selected so that the boundary would not affect the pattern formation of BB or CSK. We also think that boundary conditions are very important in to discuss pattern formation across a tissue. However, the detailed mechanism, such as how it affects the cell, is unknown (as mentioned in this manuscript). Thus, model exploration was performed excluding the unknown boundary effect.

17. The BB alignment through PCP can be better explained. Why is changing the boundary conditions significant? In Fig 5, why are the boundary conditions made to change suddenly? It seems from the snapshots 3 and 4 in Fig 5 that the transmission of polarity of CSKs takes place very quickly over the length of the tissue. If one is to get a better idea of what is happening, perhaps the system size should be increased along the length of the tissue. If the authors plan to address this in some following work, it should be mentioned.

Asymmetric localization of cortex microtubules appears in later stage as reported in Vladar et al. (Curr Biol., 2012) [19] and is dependent on biased distribution of PCP proteins. These cortical microtubules are tyrosinated and are distinguished from those running between BBs, thus we incorporated the effect as boundary condition in our model. Details of how the appearance of the cortex MTs is regulated is not clear, thus in this study we only demonstrate possible mechanism how PCP proteins can regulate direction of BBs alignment. Of course, there are ways to improve this condition, but more experimental knowledge is required. We added some sentences for explaining them (see also added sentences in DISCUSSION).

18. Newman boundary conditions should be changed to Neumann boundary conditions.

We apologize for this careless mistake. We have corrected it.

Again, we are grateful for your careful reading and useful comments and suggestions. We hope that the revision of our manuscript meets all your comments and suggestions.
